# Genome-wide analysis of the *AREB/ABF* gene lineage in land plants and functional analysis of *TaABF3* in *Arabidopsis*

**DOI:** 10.1186/s12870-020-02783-9

**Published:** 2020-12-10

**Authors:** Fangfang Li, Fangming Mei, Yifang Zhang, Shumin Li, Zhensheng Kang, Hude Mao

**Affiliations:** State Key Laboratory of Crop Stress Biology for Arid Areas and College of Plant Protection, Northwest A&F University, Yangling, Shaanxi 712100 People’s Republic of China

**Keywords:** *ABFs*, Land plants, Phylogenetic relationship, Expression analysis, *TaABFs*, Drought stress

## Abstract

**Background:**

Previous studies have shown that ABFs (abscisic acid-responsive transcription factors) are important ABA-signaling components that participate in abiotic stress response. However, little is known about the function of ABFs in *Triticum aestivum*. In addition, although various ABFs have been identified in other species, the phylogenetic relationship between ABF transcription factors has not been systemically investigated in land plants.

**Results:**

In this study, we systemically collected ABFs from land plants and analyzed the phylogenetic relationship of these *ABF* genes. The *ABF* genes are present in all the land plants we investigated, including moss, lycophyte, monocots, and eudicots. Furthermore, these *ABF* genes are phylogenetically divided into seven subgroups, differentiations that are supported by variation in the gene structure, protein properties, and motif patterns. We further demonstrated that the expression of *ABF* genes varies among different tissues and developmental stages, and are induced by one or more environmental stresses. Furthermore, we found that three wheat *ABFs* (*TaABF1*, *TaABF2,* and *TaABF3*) were significantly induced by drought stress. Compared with wild-type (WT) plants, transgenic *Arabidopsis* plants overexpressing *TaABF3* displayed enhanced drought tolerance.

**Conclusions:**

These results provide important ground work for understanding the phylogenetic relationships between plant *ABF* genes. Our results also indicate that *TaABFs* may participate in regulating plant response to abiotic stresses.

**Supplementary Information:**

The online version contains supplementary material available at 10.1186/s12870-020-02783-9.

## Background

Drought is a major environmental stressor that affects plant growth, survival, distribution, and productivity. Plants have evolved complex mechanisms in molecular, cellular, and physiological processes to respond to environmental stresses in order to survive [1]. Stressful conditions induce the production of stress response genes in plants [2, 3]. The phytohormone abscisic acid (ABA) is an important hormone that regulates some critical biological processes in plants, such as stomatal movement, adaptation to drought stress, and seed germination [4–7]. The endogenous ABA is produced when plants encounter adverse environmental stresses such as prolonged periods of osmotic stress. Several stress-responsive genes were expressed due to these increased ABA levels. Additional research indicates that many stress-responsive genes can also be induced by the exogenous application of ABA [2, 7–9].

ABA detects stress in a unique way and acts as an endogenous messenger in plant cells by inducing a double-negative regulatory pathway where ABA is bound to the ABA receptors RCARs/PYR1/PYLs, forming the complex that provides an active site for the PP2Cs. This inhibits the ability of PP2C to act as a negative regulator of the pathway, leading to the induction of SnRK2 as a positive regulator of downstream signalling and subsequent phosphorylation of the target proteins [10, 11]. Thus, in the presence of ABA, the PP2Cs are inactivated to repress SnRK2 phosphatase activity. SnRK2 could then initiate the ABA-responsive regulation pathway and activate the most significant *cis*-element ABA-responsive element (ABRE) to regulate the expression of many genes under osmotic stress conditions. Subsequently, through the yeast one-hybrid method, a subgroup of bZIP transcription factors was isolated by using ABREs as bait [12, 13]. Genes of this subgroup of bZIP transcription factors primarily participated in osmotic stress response by regulating stress-related genes. In *Arabidopsis*, nine group-A bZIP proteins were found as homologs of AREB/ABFs, and phylogenetically divided into two subfamilies, the AREB/ABF subfamily (ABF1, ABF2/AREB1, ABF3 and ABF4/AREB2) and the ABI5/AtDPBF subfamily (AtDPBF1/ABI5, AtDPBF2, AtDPBF3/AREB3 and AtDPBF4/EEL) [14]. ABF/AREB family members have four conserved domains, including two located in the C-terminus (which includes a highly conserved bZIP domain and a C4 domain) and three located in the N-terminus (which include C1, C2 and C3 domains) [15].

To date, all of these *AREB/ABF* genes in *Arabidopsis* have been functionally characterized. These four genes (*ABF1*, *ABF2/AREB1*, *ABF3* and *ABF4/AREB2*) are primarily expressed in vegetative tissues [12, 13, 16, 17]. In addition, the induced *ABF1* expression changes in response to abiotic stress are minimal [18], while *ABF2/AREB1*, *ABF3* and *ABF4/AREB2* are significantly up-regulated under ABA and osmotic stresses [12–14, 17–20]. Ectopic expression of these four genes in *Arabidopsis* showed that *ABF1* is a functional homolog of *ABF2/AREB1*, *ABF3* and *ABF4/AREB2*; and *ABF2/AREB1*, *ABF3* and *ABF4/AREB2* are the core ABA signaling components responding to abiotic stresses [16–18, 20, 21]. Moreover, the *areb1areb2abf3* triple mutant and *areb1areb2abf3abf1* quadruple mutant showed increased drought sensitivity and decreased ABA sensitivity by impairing the expression of ABA and osmotic stress-responsive genes [18, 22]. Additionally, the overexpression of many *AREB/ABFs* in various species have been shown to confer increased tolerance to osmotic stress [23–27]. Several studies have reported that *AREB/ABF* transgenic agricultural plants showed substantial increases in drought tolerance with little or no effect on growth [23, 25, 26].

Bread wheat (*Triticum aestivum* L.) is the most widely cultivated crop on earth, accounting for approximately one-fifth of the total calories consumed by humans [28]. Consequently, wheat yields and production affect the global economy. However, its productivity is frequently hampered by water scarcity, making improved drought tolerance an important goal of many breeding programs. Although several studies have demonstrated the importance of ABFs in response to abiotic stresses, our knowledge of ABFs in wheat is still very limited. In this study, we systemically described the characteristics of plant ABFs, including gene members, phylogenetic relationships, gene structures, protein structural similarities and differences, and gene expression. We performed additional functional analyses of the wheat *ABF* gene *TaABF3* by investigating drought tolerance in transgenic *Arabidopsis* plants. Our results provide an important framework for understanding the phylogenetic relationship between plant *ABF* genes and deepens our understanding of the function and mechanism of wheat *ABF* genes in responses to drought stress.

## Results

### Identification and analysis of AREB/ABF family in plants

Based on the 34 genomes listed in the Phytozome database, we performed a genome-wide BLAST search using *Arabidopsis* ABF1, AREB1/ABF2, AREB2/ABF4, and ABF3 amino acid sequences. We found the candidate ABFs in only 29 land plants, including moss, lycophyte, monocots, and eudicots. Among the ABF sequences we identified, some proteins had shorter amino acid residues (fewer than 200 amino acids). These short sequences were eliminated from subsequent analyses. In the end, 190 ABF-like sequences were collected for further analysis. We subjected these 190 protein sequences to SMART and Pfam analyses, and all of them were classified into the protein family containing bZIP domains (Pfam: 00170).

**Fig. 1:**
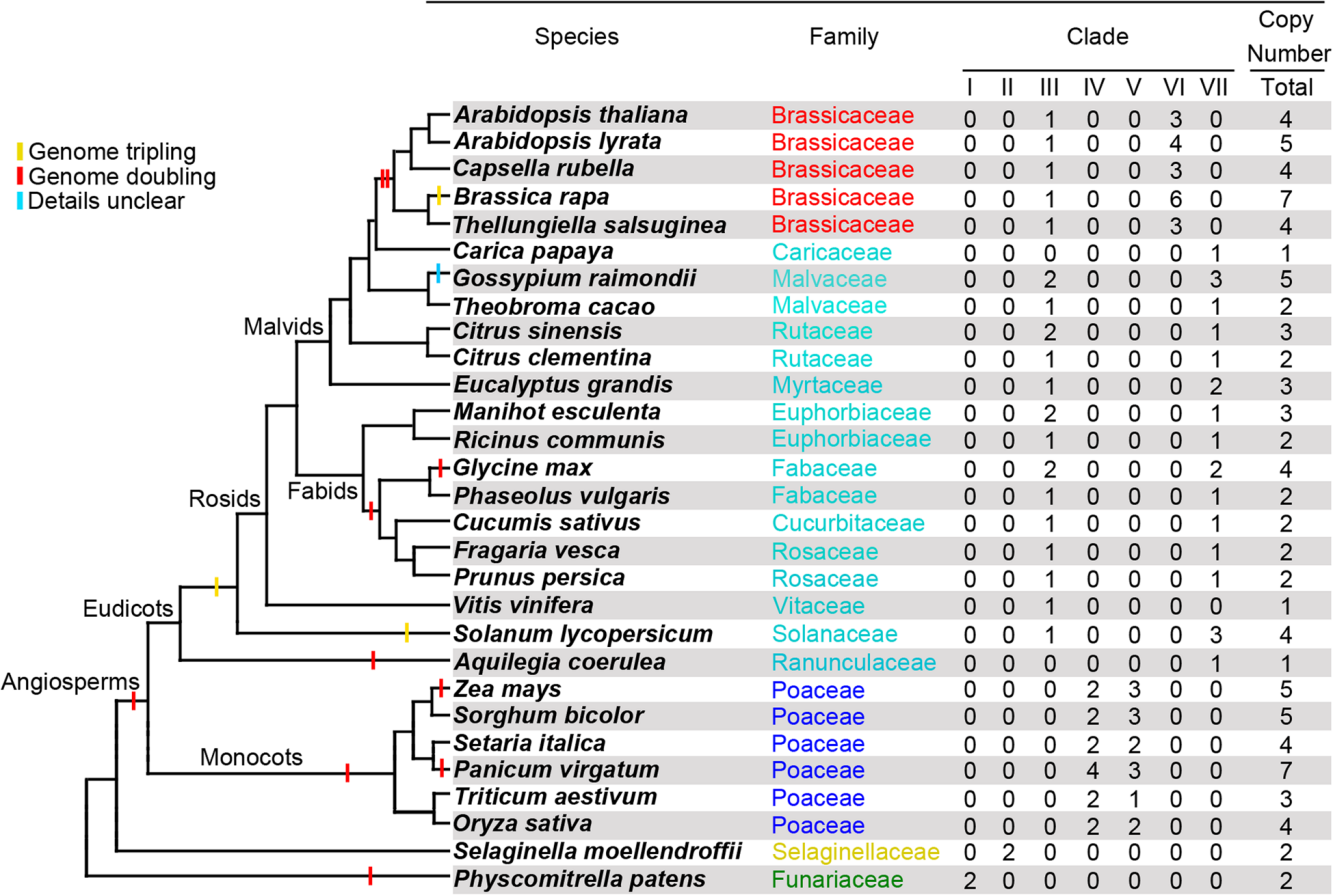
Number of *ABF* paralogs in each species and their clade distributions. The species tree is based on information in Phytozome (http://www.phytozome.net). The star on the branch point within eudicot species indicates the divergence point between a basal eudicot (*A. coerulea*) and the core eudicots

Previous studies have reported that the plant group-A bZIP family proteins can be phylogenetically clustered into two major groups, the AREB/ABF and the ABI5/AtDPBF subfamilies [14]. As such, we constructed a maximum likelihood (ML) tree, using 190 full-length ABF-like gene sequences (Additional file 1: Figure S1). Our results show that these ABF-like sequences are divided into two major clades, designated as group A and B, each having 95 identified sequences. Group A contains all experimentally characterized AREB/ABFs, including *Arabidopsis*, *Thellungiella salsuginea*, and rice ABFs [14, 15]. According to previously characterized genes, such as ABI5/AtDPBF1, AtDPBF2, AREB3/AtDPBF3 and EEL/AtDPBF4 [14], group B was classified as ABI5/AtDPBF subfamilies. Therefore, group A sequences are designated ABF and were included for further analyses (Additional file 2: Table S1). The number of ABFs in each species is shown in Fig. 1. In summary, the moss *Physcomitrella patens* and the lycophyte *Selaginella moellendorffii* have two copies of the ABFs. In monocots, all species contain only four copies of ABFs, with the exception of wheat, maize and *Panicum virgatum*. The ABF copy number differed, from one to seven, in eudicots. This indicates that several duplication incidents took place. The quantity of ABF paralogs in rice, *Arabidopsis*, and *Thellungiella* observed by this study are in line with previous research [14, 15].

We further analyzed protein length, molecular mass, and the pI values of 95 ABF proteins (Additional file 2: Table S1). According to our results, the length and molecular mass of ABFs ranged from 254 to 485 amino acid residues, and 27.81 to 52.95 kD, with a mean of 389 amino acid residues and 42.16 kD. *Aquilegia coerulea*_ABF1 is the longest and largest ABF (485 amino acid residues and 52.95 kD), while *Citrus sinensis*_ABF3 is the shortest and smallest ABF (254 amino acid residues and 27.81 kD). *Zea mays*_ABF5 has the lowest pI value, with 5.44, while *Citrus sinensis*_ABF3 has the highest value, with 10.42. ABFs in clades III, VI and VII have very close pI values, while the pI values of ABFs in clades I, II, IV, and V varied widely. Interestingly, ABFs from clade V displayed a tendency to maintain acidic pI values, with an average of 6.97, while more alkaline pI values (greater than 7) were observed in 83 out of 95 ABFs belonging to other clades (Table 1; Additional file 2: Table S1).

**Table 1:** Summary of ABF protein properties

Clade	Total No. of ABFs	Protein length (aa)	Molecular Mass (KD)	Theoretical pI	No. of ABFs with PI > 7
I	2	407 ± 60.81	42.45 ± 6.76	8.60 ± 3.03	2
II	2	328 ± 101.82	35.39 ± 7.72	8.05 ± 1.61	2
III	23	394 ± 55.16	42.51 ± 5.82	9.63 ± 0.34	23
IV	14	343 ± 13.91	36.99 ± 1.51	8.69 ± 1.32	12
V	15	344 ± 20.08	36.67 ± 2.21	6.97 ± 1.03	5
VI	19	417 ± 32.06	45.62 ± 3.48	9.10 ± 0.66	18
VII	21	421 ± 24.87	46.01 ± 2.68	9.40 ± 0.63	21
Minimum	–	254	27.81	5.44	–
Maximum	–	485	52.95	10.42	–

### Phylogenetic and structural analysis of plant ABFs

**Fig. 2:**
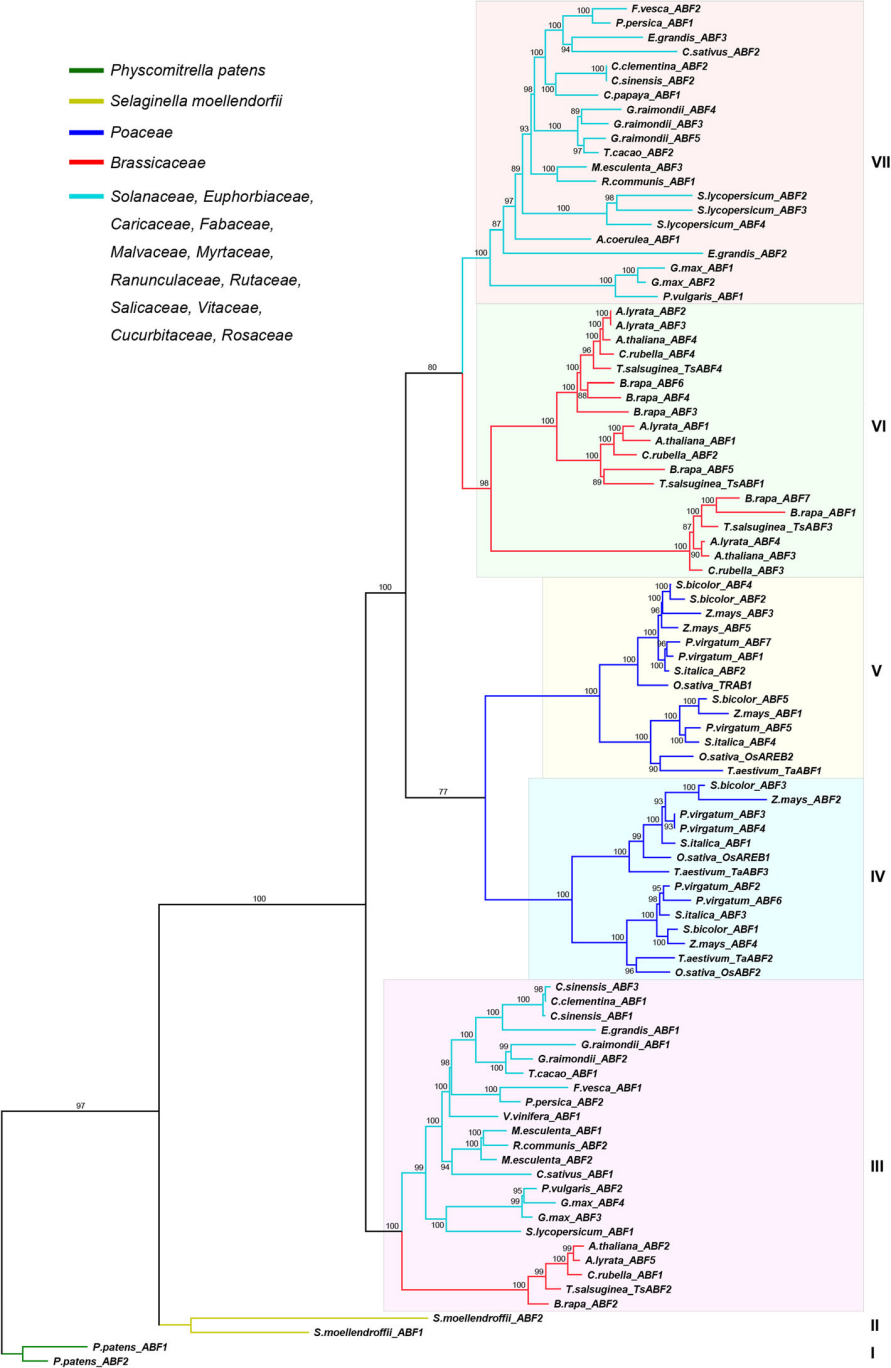
Phylogenetic relationship of plant ABF genes. ABFs are grouped into seven distinct clades (I-VII). Numbers above branches represent the support values

In order to better understand the evolutionary relationship of AREB/ABF members in land plants, we further constructed an ML tree using full-length protein sequences of 95 ABFs. According to support values (85% or greater) of the phylogenetic tree, ABFs can be divided into seven clades (clades I to VII) (Fig. 1; Fig. 2). Inside the phylogenetic tree, ABFs from the moss *Physcomitrella patens* and the lycophyte *Selaginella moellendorffii* form two independent clades, assigned as clades I (*P.patens*_*ABFs*) to II (*S.moellendorffii*_*ABFs*). The monocots can be placed into the next two clades, IV and V. The eudicots can be divided into three clades: III, VI, and VII.

It is worth mentioning that the phylogenetic tree (Fig. 2) aligns with the species tree shown in Phytozome (Fig. 1) with the ABFs from moss (*Physcomitrella patens*) and lycophyte (*Selaginella moellendorffii*) forming the two basal lineages of land plants. Monocot and eudicot ABFs are closer on the phylogenetic tree and form two monophyletic clades. To further investigate the accuracy of the ABF phylogenetic tree, we analyzed the exon/intron organization for each individual gene (Additional file 3: Figure S2). Of the 95*ABFs*, one has one exon; three have two exons; four have three exons; 71 have four exons, 13 have five exons, two have six exons, and two have seven exons. Within each clade, the gene structure of *ABFs* is relatively conserved, and the adjacent *ABFs* have a similar exon/intron structure. We then investigated the intron phases of all *ABF* gene structures. There are three categories of intron phase: phase 0 intron, phase 1 intron, and phase 2 intron. Our analysis indicated that the intron phase patterns (0, 0, 0) and (0, 0, 0, 0) are the predominant patterns across 95 land plant *ABFs* (Additional file 3: Figure S2). This analysis indicated that we have constructed a phylogenetic tree of the *ABF* genes in land plants that is highly accurate.

### Motif composition and arrangement of plant ABFs

**Fig. 3:**
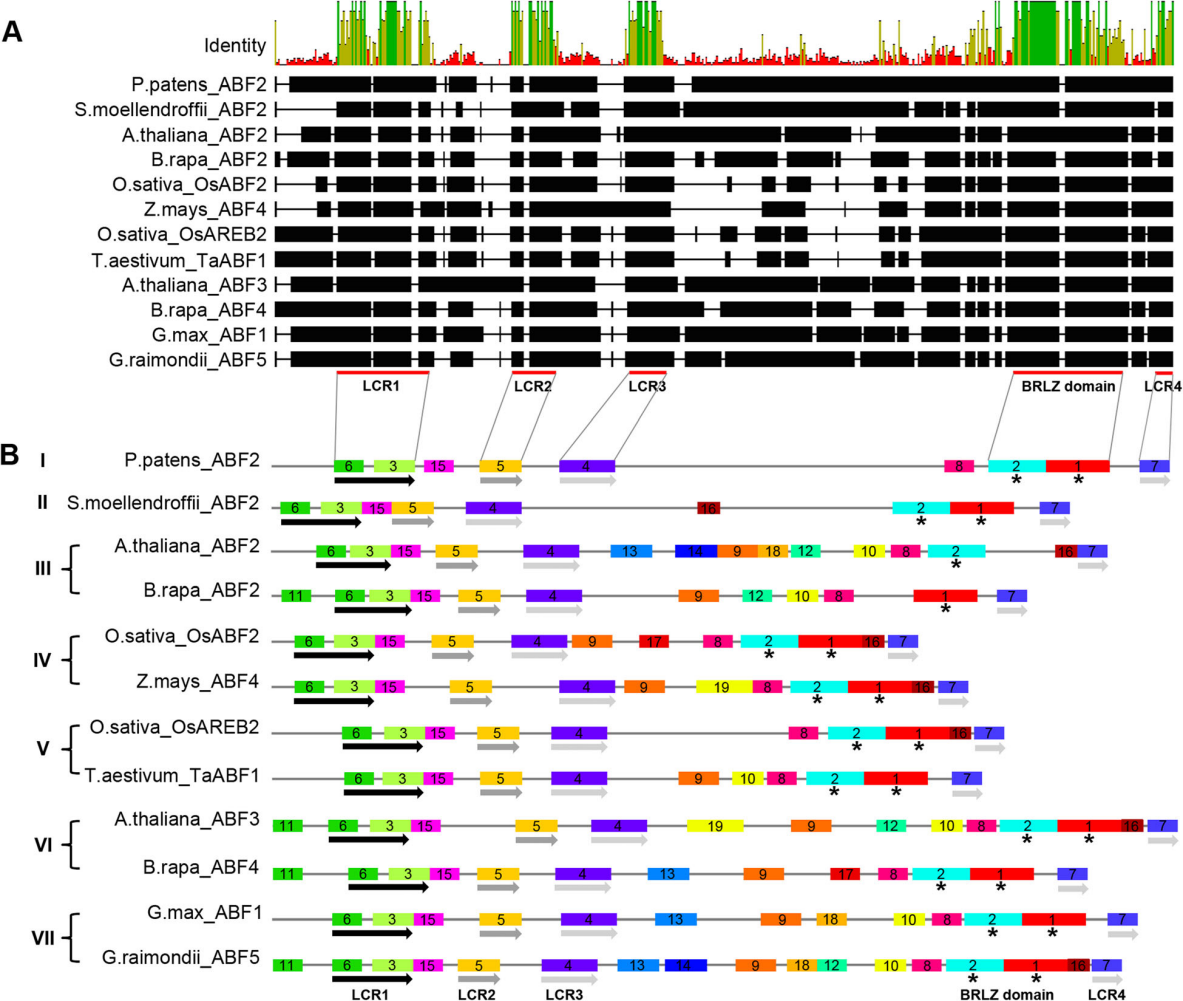
Sequemce alignment and motif composition of plant ABFs. **a** Alignment of protein sequences in representative plant ABFs. The conserved domains are annotated as red lines (LCR1-LCR4, BRLZ domain). The alignment was generated using ClustalW implemented in the Geneious software and represented as thick lines (aligned characters) and thin lines (gaps). **b** Motif patterns in representative ABFs. Motif occurrences were predicted using the MEME program, and the different colored and numbered boxes represent separate and distinct motifs. Stars indicate the BRLZ domain and four different colored arrows indicate the four conserved motifs (LCR1-LCR4)

**Fig. 4:**
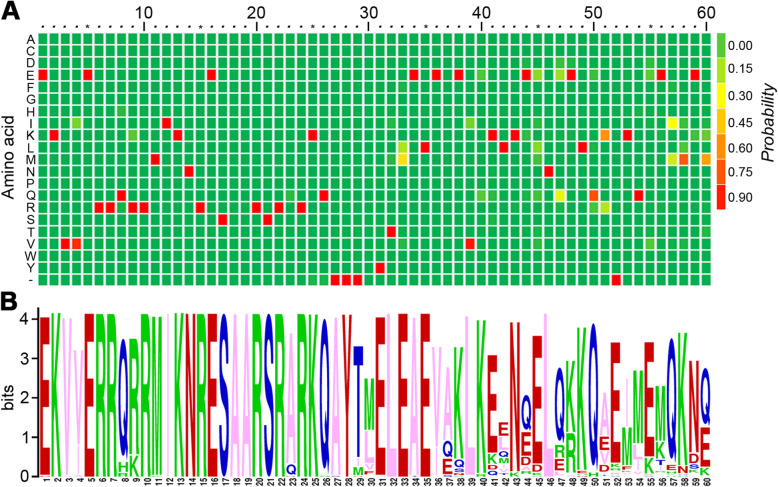
The sequences of the BRLZ domain from ABFs are conserved across land plants. **a** A heat map diagram of the protein sequence alignment of BRLZ domain from land plants. **b** ICE LOGO of the BRLZ domain in the N-terminus of ABF shows that the motif is strictly conserved in land plants

In order to better understand the phylogenetic relationships between plant ABFs, we aligned all of the ABF sequences to better identify the conserved amino acid residues. Based on the alignment, 35 amino acid residues are completely conserved in 88 ABFs (except for eight shorter ABFs). We further identified the conserved motifs in 95 plant ABFs using the SMART program. Finally, we found fiveconserved protein motifs in all ABFs, which are BRLZ domain and the other four low complexity regions (LCR 1–4; Fig. 3a; Additional file 4: Figure S3). ABFs belong to the basic-leucine zipper (bZIP) domain transcription factor family, and we found that the BRLZ domains are highly conserved in all plant sequences (Fig. 4).

However, the ability of SMART to comprehensively identify the motifs present in ABFs is limited, so we used the MEME program to identify conservation and variation in the motif arrangements among ABFs. We identified 20 distinct motifs in ABFs. The occurrences and arrangements of the motifs in ABFs from seven major clades are shown in Fig. 3b and Additional file 5: Figure S4. Among 20 motifs, 8 motifs are shared by all ABFs, which are components of the BRLZ domain (motif 1 and 2) and the other four conserved low complexity regions (motif 3 and 6 for LCR1, motif 5 for LCR2, motif 4 for LCR3, and motif 7 for LCR4). Next, we examined the non-conserved motif composition in land plant ABFs. We then split the ABFs into four regions, based on the location of the LCR motifs and the BRLZ domain (Fig. 3b): Region 1 is the part before the LCR1, Region 2 is the part between LCR1 and LCR2, Region 3 is the part between LCR3 and the BRLZ domains (there were no motifs between LCR2 and LCR3), and Region 4 is the part between the BRLZ domain and LCR4. Of these four, Regions 2 and 4 are highly conserved in plants on land (they are mainly comprised of motifs 15 and 16). Less conserved is Region 1, which is primarily comprised of motif 11 in clades III, VI, and VII. Region 3 is the most divergent region: motif 8 was observed in clades I, III, IV, V, VI, and VII; motifs 9 and 10 were found in clades III, IV, VI, and VII; motifs 12 and 17 were found in clades III, V, VI, and VII; motifs 13 and 14 were found in clades III, VI,and VII; motifs 18 were found in clades III and VII; motif 19 was found in clades IV and VI; and motif 20 was found in clade V (Fig. 3b). Taken together, the conserved and non-conserved motif patterns of plant ABFs that we identified match the pattern of clades in the phylogenetic tree.

### Expression analysis of plant *ABF* genes

To obtain the expression profiles of *Arabidopsis* ABFs, we extracted the expression data from the *Arabidopsis* eFP Browser (http://bar.utoronto.ca/efp/cgi-bin/efpWeb. cgi). We found that the expression of *Arabidopsis* ABF paralogs displayed tissue differentiation. For example, *A.thaliana_ABF1* displayed significantly higher expression in roots, and *A.thaliana_ABF2* displayed significantly higher expression in seeds, indicating that ABF paralogs have followed the trend of tissue subfunctionalization. We found that ABF paralogs in clade III (*A.thaliana_ABF2*) have higher expression levels than clade VI paralogs (*A.thaliana_ABF1*, *A.thaliana_ABF3*; Additional file 6: Figure S5A). We next investigated the expression profiles of other plant ABF genes. Our results demonstrated that soybean (*Glycine max*) and common bean (*Phaseolus vulgaris*) ABF paralogs are expressed more in leaves, roots, and flowers than in other tissues, and that ABF paralogs in clade III (*G.max_ABF3*, *P.vulgaris_ABF2*) have higher expression levels than clade VII paralogs (*G.max_ABF1*, *G.max_ABF1*, *P.vulgaris_ABF1*) (Additional file 6: Figure S5B and C). Within monocots, we studied the expression of the four ABF paralogs from rice (*Oryza sativa*), and found that *O.sativa_OsABF2* (clade III) had higher expression levels than *O.sativa_TRAB1*, *O.sativa_OsAREB1*, and *O.sativa_OsAREB2* (Additional file 6: Figure S5D). The five ABFs in maize (*Zea mays*) display similar expression patterns among tissues, except for *Z.mays_ABF3*, which is less expressed among all tissues (Additional file 6: Figure S5E). The expression divergence of plant ABFs indicated the functional differentiation of ABFs.

**Fig. 5:**
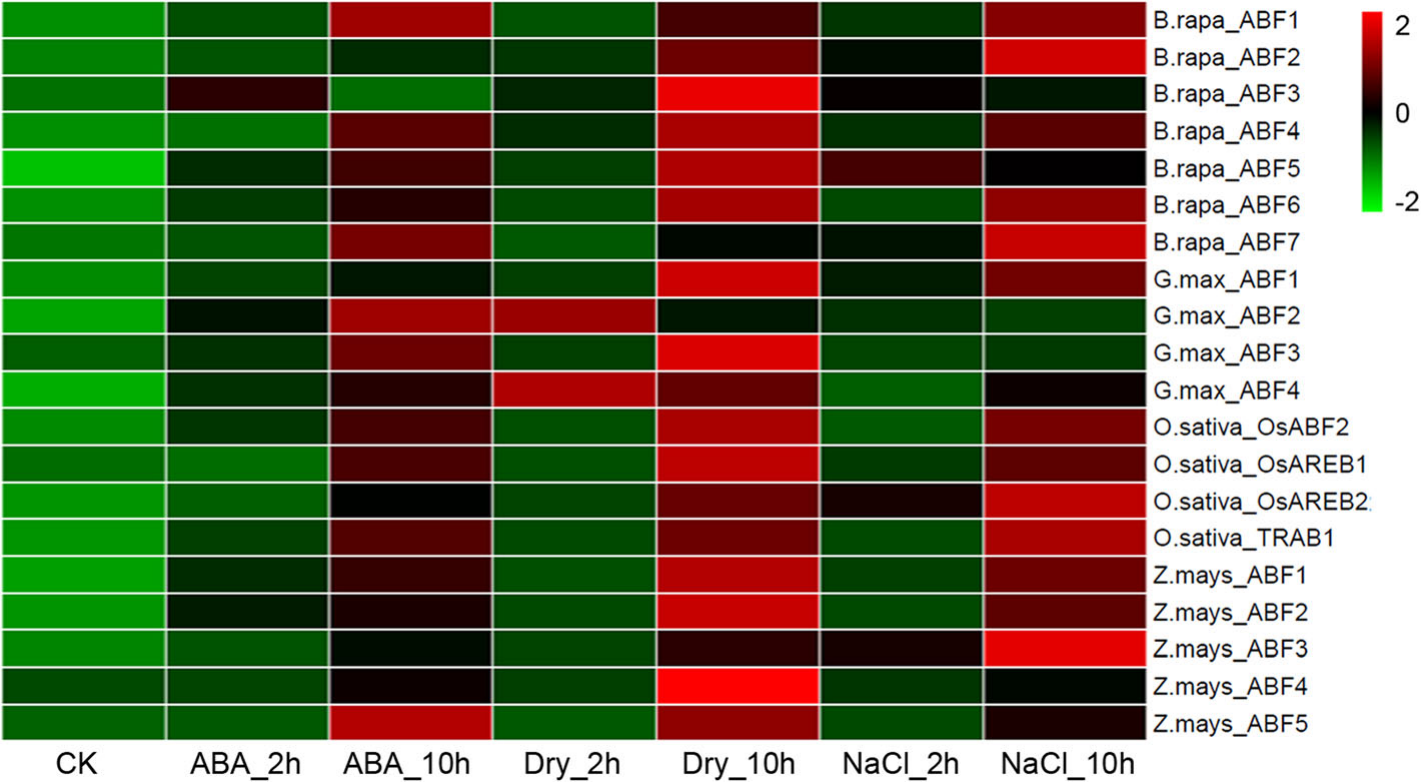
Expression profiles of representative plant *ABF* genes under different abiotic stresses. The relative expression levels of *ABFs* were determined using quantitative real-time RT-PCR in the leaves of three-leaf-stage seedlings treated with drought, NaCl, and ABA, compared with the control

We also investigated the expression of *Arabidopsis ABFs* under abiotic stresses using microarray expression data. The results showed that the expression of all *Arabidopsis* ABFs was induced by ABA, cold temperatures, drought conditions, and high salinity, but the degrees of induction differed. *A.thaliana_ABF1* was significantly induced by cold temperatures; *A.thaliana_ABF2* was significantly induced by drought conditions; *A.thaliana_ABF3* was significantly induced by ABA, drought conditions and salt; and *A.thaliana_ABF4* was significantly induced by drought conditions and salt (Additional file 7: Figure S6). We further investigated the expression of other plant *ABFs* to abiotic stresses (ABA, drought and highly salinity) using quantitative real-time PCR (qRT-PCR). From the heatmap, we found that the expression of most *ABFs* was induced by ABA, drought conditions, and high salinity. Except for *B.rapa_ABF7*, *G.max_ABF2*, and *Z.mays_ABF3*, all ABFs were significantly induced by drought conditions. ABFs are known for their importance in ABA-mediated abiotic stress responses, meaning a significant induction in the *ABF* genes might play a crucial role in plant adaptation to environmental stresses (Fig. 5).

### Molecular characterization and expression analysis of *TaABFs*

**Fig. 6:**
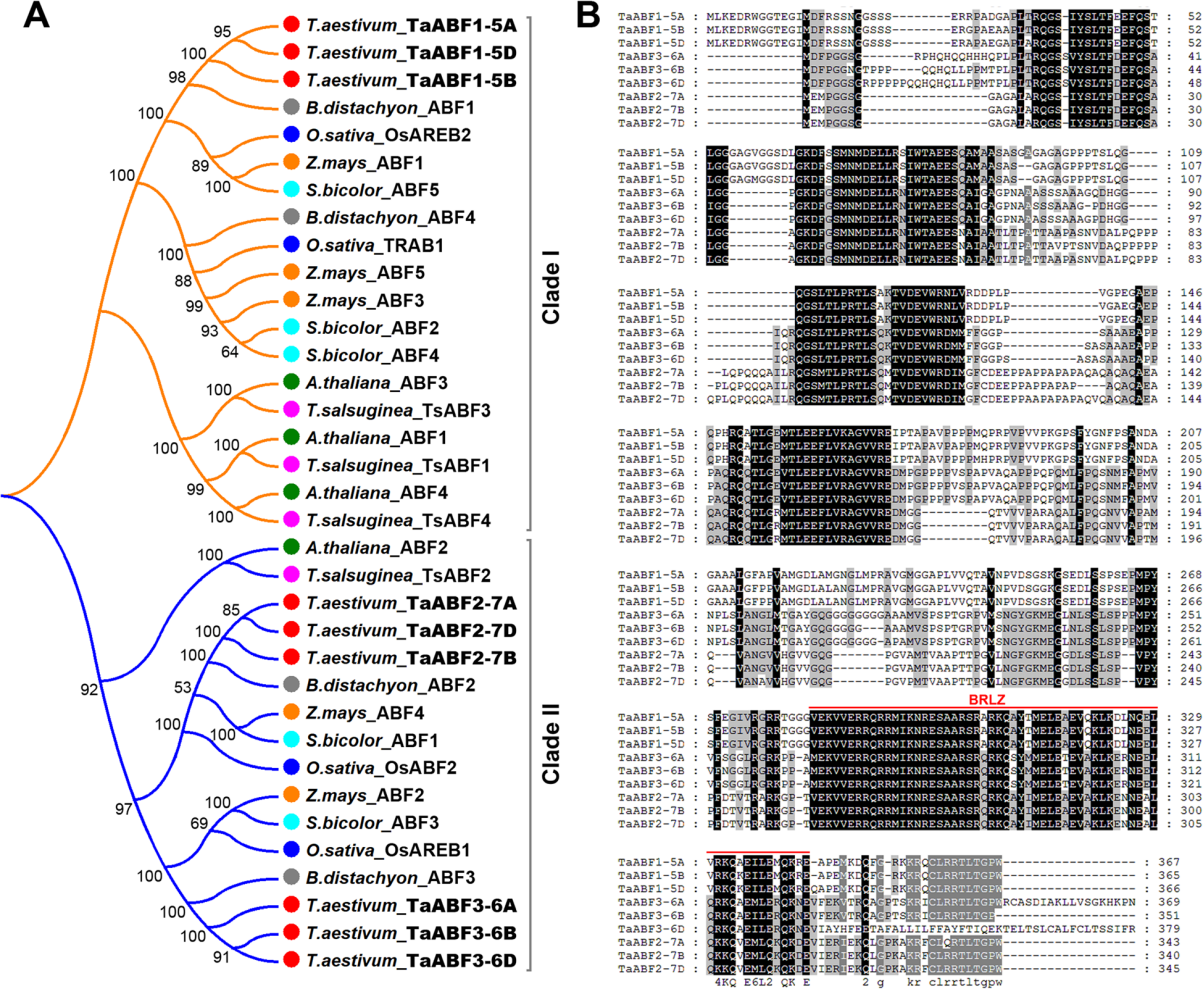
Phylogeny, subcellular localization, and expression of *TaABFs*. **a** Phylogenetic relationship between TaABFs and ABF members from other plant species. The phylogenetic tree was constructed by MEGA6.0 using the neighbor-joining method. The numbers at each node indicate the percentage of bootstrap values from 1000 replicates. **b** Protein sequence alignment of TaABFs. The locations of the highly conserved BRLZ domain was indicated by black lines

**Fig. 7:**
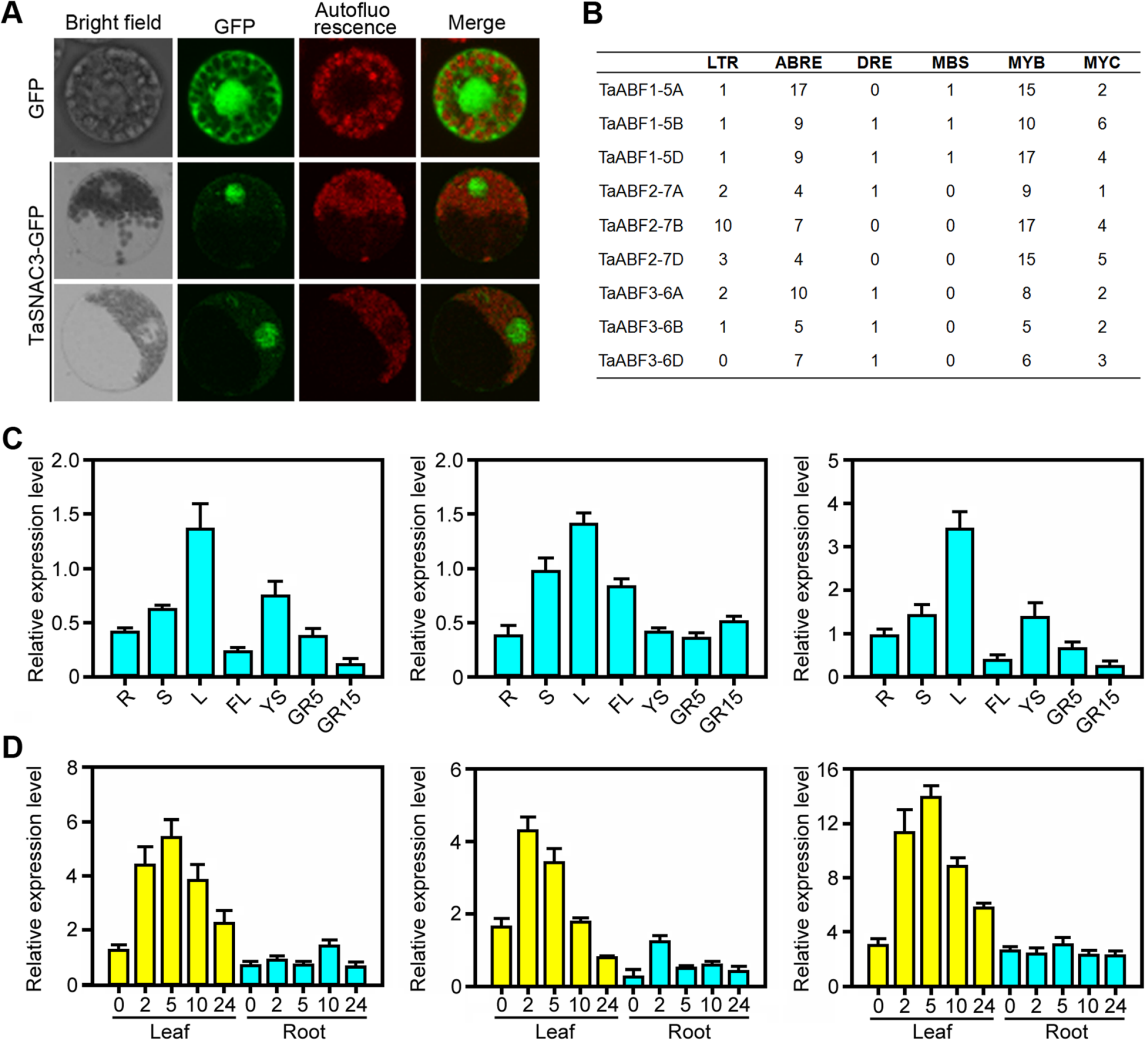
Molecular characterization and expression analysis of *TaABFs*. **a** Subcellular localization of TaABF3 protein. 35S:GFP was used as positive control. **b** Distribution of several stress-related *cis*-elements in the promoter region (~ 2.0 kb) of *TaABFs*. LTR, low temperature responsive element; ABRE, ABA-responsive element; DRE, dehydration-responsive element; MBS, MYB binding site involved in drought-inducibility; MYB, MYB recognition site; MYC, MYC recognition site. **c** The expression profiles of *TaABFs* in different tissues. R, root of wheat seedling at five-leaf stage; S, stem of wheat seedling at five-leaf stage; L, leaf of wheat seedling at five-leaf stage; FL, flag leaf at heading stage; YS5, young spike at early booting stage; GR5, grain of 5 days post-anthesis; GR15, grain of 15 days post-anthesis. **d** The expression pattern of *TaABFs* under drought stress treatment. The numbers on X axis indicate the time point subject to drought stress. The error bars indicate standard deviations derived from three independent biological experiments

Phylogenetic analyses suggest that TaABFs might serve a role in regulating abiotic stress response in wheat. We cloned three *TaABF* genes from the wheat *cv*. Chinese Spring. Each gene had three homologous components in the A, B, and D genomes of wheat; we named them *TaABF1-5A/B/D*, *TaABF2-7A/B/D*, and *TaABF3-6A/B/D*. Additional phylogenetic analyses indicated that TaABF1 was most closely related to the rice OsAREB2, TaABF2 was most closely related to the rice OsABF2, and TaABF3 was most closely related to the rice OsAREB1 (Fig. 6a). An analysis of the protein sequence revealed that TaABFs displayed 55–98% sequence similarity (Fig. 6b). We then analyzed the subcellular localization of TaABF3, first constructing the expression cassette and fusing TaABF3 with the GFP protein. The fused proteins were then transiently expressed in *Arabidopsis* protoplasts. We used fluorescence microscopy to analyze and reveal that the TaABF3-GFP fusion proteins were exclusively localized in the nucleus in the transformed cells, while the control GFP was uniformly distributed throughout the cell (Fig. 7a). These results confirmed that TaABF3 is a nuclear-localized protein.

To examine the expression pattern of *TaABFs*, we first identified the *cis*-element in its region of promotion, which was ~ 2 kb upstream of the transcription initiation codon, finding a number of *cis*-acting elements related to stress response in the promoter of *TaABF*s. This includes LTR (low temperature-responsive element), MYB (MYB recognition site), MYC (MYC recognition site), MBS (MYB binding site involved in drought-inducibility), ABRE (ABA-responsive element), and DRE (Dehydration-responsive element) (Fig. 7b). In order to better understand the role that TaABFs play in response to drought conditions, we executed quantitative real-time PCR (qRT-PCR) on RNA taken from various tissues and conditions of drought. Considering the highly sequence similarity of wheat homeologous genes, the PCR primers were designed to amplify the conserved locus of three *TaABF* homeologs; for example, the relative expression level of *TaABF1* represents the combined expression of all three *TaABF* homeologs (*TaABF1-5A*, *TaABF1-5B* and *TaABF1-5D*). The results demonstrated that TaABFs were found in higher levels in the leaves of the seedlings (Fig. 7c) and that under drought stress conditions, all *TaABFs* in wheat leaves were up-regulated (Fig. 7d).

### Overexpression of *TaABF3* confers drought tolerance in *Arabidopsis*

**Fig. 8:**
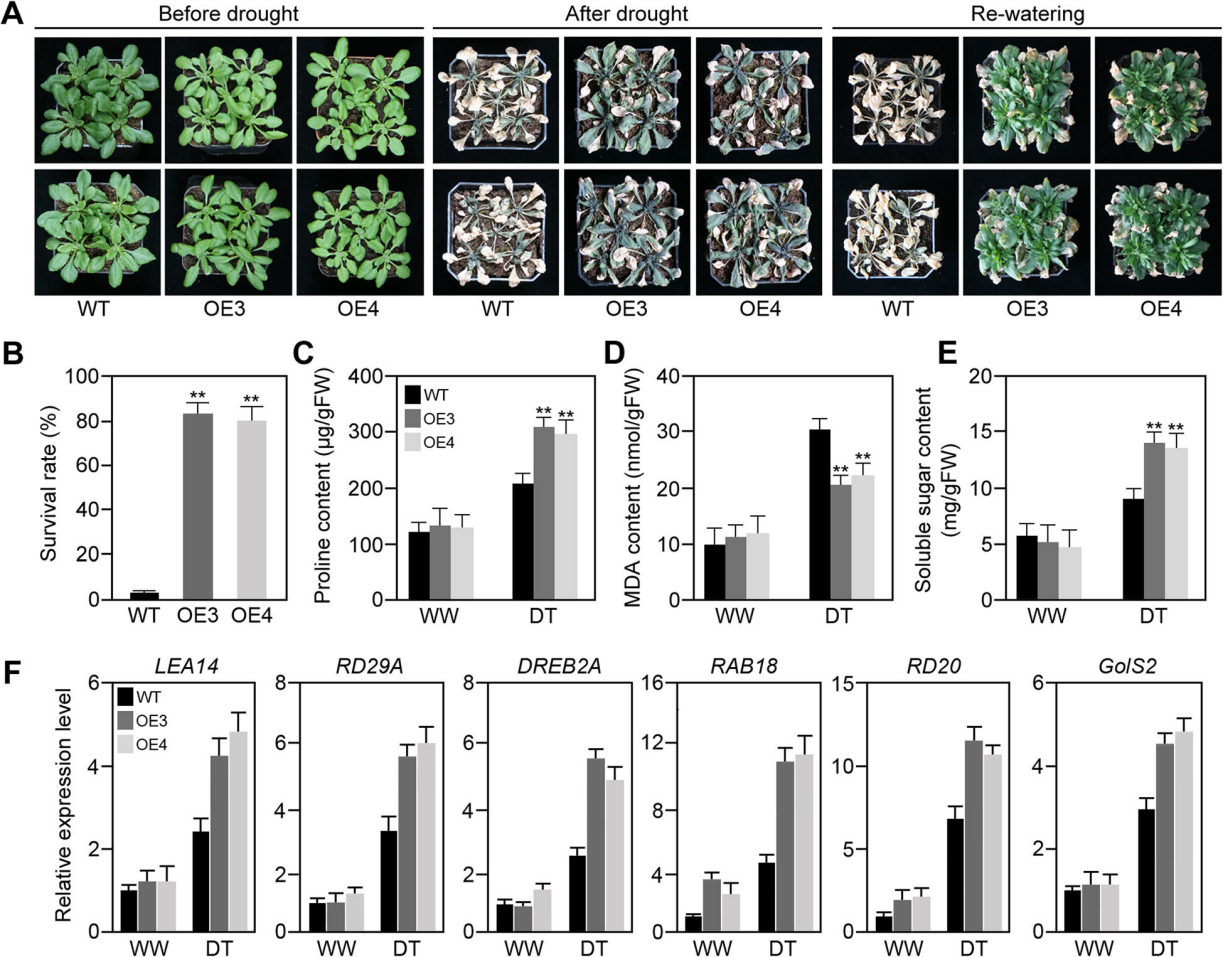
Drought tolerance assay of *TaABF3* overexpression transgenic *Arabidopsis* plants. **a** Performance of *TaABF3* overexpression *Arabidopsis* plants under drought stress. **b** Statistical analysis of survival rates after the drought stress treatment. The average survival rates and standard errors were calculated based on data obtained from three independent experiments. **c**-**e** Proline contents (**c**), malondialdehyde (MDA) contents (**d**) and soluble sugar contents (**e**) of the WT and transgenic lines before and after drought treatment. F qRT-PCR analysis of the selected marker genes involved in water response. *TaActin* was used as the internal control. WW, well-watered; DT, drought stress

To better understand how TaABFs function in plant abiotic stress tolerance, we generated *35S::TaABF3-GFP* transgenic *Arabidopsis* lines. We then selected three independent transgenic lines for *35S::TaABF3-GFP* transgenic *Arabidopsis* that exhibited higher expression levels of *TaABF3* in order to further analyze their response to drought stress (Additional file 8: Figure S7). We then compared the drought tolerance of transgenic and vector-transformed (WT) plants. We grew WT and each *35S::TaABF3-GFP* transgenic plants for 3 weeks in soil before withholding water for ~14d. After the drought treatment and 6 days of re-watering, ~ 65–75% of the transgenic plants survived, while only ~ 8% of the WT plants survived (Fig. 8a and b).

We next assayed the proline contents, malondialdehyde (MDA) contents, and the soluble sugar contents in *35S::TaABF3-GFP* transgenic and WT plants (Fig. 8c-e). Our results showed that in transgenic lines the proline contents and the soluble sugar contents were significantly higher and the MDA contents were significantly lower than in WT under both well-watered and drought conditions. We also detected the expression of several well-known drought-responsive genes in the transgenic lines, including *Arabidopsis-*homologous *LEA14* [29], *RD29A* [30], *DREB2A* [31], *RAB18* [32], *RD20* [33], and *GolS2* [34]. These results showed that all of these genes were up-regulated in *35S::TaABF3-GFP* transgenic lines (Fig. 8f). Collectively, these findings indicate that the overexpression of *TaABF3* in *Arabidopsis* could enhance the drought tolerance of transgenic plants.

## Discussion

Transcription factors (TFs) are a group of regulatory proteins that regulate gene expression by binding to specific *cis*-acting elements in the promoters of target genes [35]. Despite the fact that many studies have revealed the crucial role of AREB/ABF TFs in response to abiotic stresses [16–18], our knowledge of ABFs is still limited. Previous studies have primarily focused on studying the function of ABF/AREB proteins, whereas phylogenetic studies of ABFs are restricted to some model plants, such as *Physcomitrella patens*, *Selaginella moellendorffii*, *Arabidopsis*, and rice [14]. To advance our understanding of the involvement of ABFs in stress response and other biological processes, it is essential to first understand their evolution and diversity. In this study, we collected most land plant group-A bZIP TFs from available genome databases (Fig. 1; Additional file 2: Table S1) and performed phylogenetic analyses with full coding sequences. This allowed us to identify the ABF clade within the bZIP TFs (Fig. 2), the intron/exon structure of genes (Additional file 3: Figure S2), and the characteristic protein domains (Fig. 3; Fig. 4; Additional file 4: Figure S3; Additional file 5: Figure S4). We next extracted expression profiles of selected plants from a public expression database and explored the functional differences of paralog genes during land plant evolution (Fig. 5; Additional file 6: Figure S5; Additional file 7: Figure S6). In addition, we systemically investigated the function of target genes of TaABF3 (Fig. 6; Fig. 7; Fig. 8). The goal of our study was to provide an overall picture of plant ABFs and deepen our knowledge of the function and mechanism of wheat *ABF* genes when responding to abiotic stresses.

At the protein level, ABFs in land plants share many of the same structural features, all ABFs have four conserved LCR motifs, and one BRLZ domain (Fig. 3a; Additional file 4: Figure S3). However, the differences of ABF proteins are also existed. For example, the protein structure between the LCR3 motif and the BRLZ domain exhibit the highly variable (Fig. 3b). This region requires additional research to further elucidate the differences in structure and function between the ABFs in land plants from various clades.

A robust phylogenetic tree is essential for tracing the evolutionary history of *ABF* genes. As sequencing techniques have advanced, increasing amounts of plant genomes have been sequenced and released. In this study, we surveyed 34 different plant genomes and collected 95 ABF genes. With the exception of algae, ABF candidates exist in all land plants, including lowland plants (a moss and a lycophyte) and highland plants (monocots and eudicots). It is increasingly apparent that gene families present in embryophytes (land plants) and absent from sequenced chlorophyte genomes have their origins in the kind of algae from which the ancestral land plant evolved. This indicates that the *ABF* gene family originated during the evolution of the algal to land plants. Previous studies favor the single-origin theory of land plants, originating from charophycean green algae [36, 37]. Moving from an aqueous to a gaseous environment subjects various plants to different physical conditions, which results in particular changes to their structure and physiology. Significant metabolic pathways, involving flavenoids, lignins, plant hormones, and cutins from vascular plants come from existing structures of the primary metabolism in charophycean algae [36]. During this process, various families of genes evolved and helped land plants to adapt to challenging new environmental conditions, which included abiotic stressors. It is possible that *ABF* genes could be induced by several abiotic stresses, participating in stress response to abiotic factors [16–18]. This evolution of ABFs could have played an important role in allowing plants to adapt to conditions on land.

Our analysis of the phylogenetic relationship demonstrated that the ABF gene family underwent two changes that led to the seven distinct subfamilies (Fig. 2). The first instance happened after *Selaginella moellendorffii* and *Physcomitrella patens* diverged from a common ancestor, that of seed plants. This occurrence is consistent with the known patterns of divergence in land plants, where *Selaginella moellendorffii* and *Physcomitrella patens* are the precursors of the seed plants. Following this event, the family of ABF genes could have been limited to their historical functions. However, our phylogenetic tree shows that the second instance of duplication that resulted in lineages similar to ABF happened in seed plants. Prior research has found that a whole-genome duplication (WGD) event that occurred in an ancestor of extant angiosperms produced exact copies of each gene [38, 39]. Monocots have seen many instances of WGD throughout their history, which are surely responsible for the high instance of *ABF* genes (Fig. 1) [40]. All of the ABFs in monocots were found in clades IV and V, which is evidence of a duplication event early in the evolutionary history of monocots. In contrast, eudicot ABFs were all found in clades III, VI, and VII. Members of clade III are paralogs of *Arabidopsis ABF2*, while members of clade VI are paralogs of *ABF1*, *ABF3*, and *ABF4.* Members of clade VII are more similar to members of clade VI but are not found in the paralogs of *Arabidopsis*. All of this is evidence of the functional similarity of ABFs, while the differences between the clades are indicative of functional differentiation between the clades. Recent research has shown that ABFs are involved in ABA signaling when responding to abiotic stressors [16–27], while the functional differences between *ABF* genes remain scarce. One study found that ABF2, ABF3, and ABF4 play important roles when regulating the mediation of ABA-triggered Chl degradation as well as leaf senescence in *Arabidopsis* [41]. This demonstrates evolutionary divergence in the functionality of ABFs,but in order to understand the practical differences within the lineage of ABFs, further research is required.

The responsiveness of *ABF* genes to abiotic stress strongly suggests that they serve roles in adapting to changing environmental conditions. Our qRT-PCR analyses further revealed that all *TaABF* genes were induced by drought stress (Fig. 7). To investigate the role of *TaABF* genes in the abiotic stress response, *TaABF3* was transformed into *Arabidopsis*, and its overexpression was confirmed by RT-PCR (Additional file 8: Figure S7). The transgenic plants showed significantly improved drought and salt tolerance compared to WT plants (Fig. 8). Consistently, several stress-responsive genes, including *LEA14*, *RD29A*, *DREB2A*, *RAB18*,*RD20*, and *GolS2* were found to be significantly up-regulated in *TaABF3* transgenic *Arabidopsis* under drought stress (Fig. 8). This research strongly indicates that *TaABF3* increases the tolerance of transgenic *Arabidopsis* plants to drought conditions. Prior research has shown that an overexpression of TF genes can slow the growth of transgenic plants [42–45]. We also closely monitored the growth and morphological features of *TaABF3* transgenic *Arabidopsis* plants, finding that transgenic plants exhibited a slight reduction in the size of rosette leaves (Fig. 8).

## Conclusions

In summary, our study provides a comprehensive analysis of the plant ABF genes, include phylogenetic relationships, gene structures, protein structures and properties, and expression profiles. Phylogenetic analysis combined with gene structure and motif composition clustered the plant ABFs into seven distinct clades. In addition, expression analyses demonstrate that plant ABFs have extensively induced by abiotic stress. Further functional analysis of *TaABF3* trangenic *Arabidopsis* showed that they could confers drought tolerance in plants. Our results will help elucidate the functions of the AREB/ABF lineage in plants, and providing clues for the identification of candidate genes involved in abiotic stress responses in plants.

## Methods

### Plant materials and stress treatments

After subjecting four plant species (*Brassica rapa*, *Glycine max*, *Oryza sativa*, and *Zea mays*) to different stress conditions, we assayed the expression of *ABFs*. We obtained *Brassica rapa* cv. ZS11, *Glycine max* cv. Jidou-7, *Oryza sativa* cv. Nipponbare, and *Zea mays* cv. B73 from Northwest A&F University, though these strains also could have been acquired from the Chinese Crop Germplasm Resources Information System (http://www.cgris.net/zhongzhidinggou/index.php). Growth conditions and the application of stress conditions proceeded according to the following: we germinated 1‰ (v/v) Topsin-M sterilized seeds at 25 °C for 3 days on wet filter paper. The germinated seeds were then grown hydroponically, with Hoagland nutrient solution, under a 16 h light/8 h dark photoperiod in an artificially controlled climate chamber at 25 °C. The abscisic acid (ABA) was then applied to the cultivated seedlings, followed by high salinity conditions, and finally, drought conditions. The three-week-old seedlings were placed into a 200 mmol/L NaCl solution for the high-salinity treatment, into a 100 μmol/L ABA culture solution for the ABA treatment, and on a clean bench for the drought treatment (where they were dehydrated at 25 °C and relative humidity of 40–60%). The whole seedlings were collected 0, 2, and 10 h after subjecting them to stress conditions. We collected a minimum of five seedlings from each plant species at each time point, while each experiment was performed three times. All samples were subsequently frozen in liquid nitrogen and refrigerated at − 80 °C prior to RNA extraction.

### *ABF* genes identification

For *Arabidopsis*, ABFs (AREB1/ABF2, AREB2/ABF4, ABF1 and ABF3) were used to conduct a TBLASTN query in the Phytozome databases (http://www.phytozome.net/). We used 34 plant species, including algae (*Chlamydomonas reinhardtii*, *Coccomyxa subellipsoidea* C-169, *Micromonas pusilla* CCMP1545, *Ostreococcus lucimarinus*, and *Volvox carteri*), moss (*Physcomitrella patens)*, lycophyte (*Selaginella moellendorffii)*, monocots (*Triticum aestivum*, *Oryza sativa*, *Panicum virgatum*, *Sorghum bicolor*, *Setaria italica*, and *Zea mays*), and eudicots (*Aquilegia coerulea*, *Arabidopsis lyrata*, *Arabidopsis thaliana*, *Brassica rapa*, *Capsella rubella*, *Carica papaya*, *Citrus clementina*, *Citrus sinensis*, *Cucumis sativus*, *Eucalyptus grandis*, *Fragaria vesca*, *Glycine max*, *Gossypium raimondii*, *Manihot esculenta*, *Mimulus guttatus*, *Phaseolus vulgaris*, *Prunus persica*, *Ricinus communis*, *Solanum lycopersicum*, *Thellungiella salsuginea*, *Theobroma cacao*, and *Vitis vinifera*). The amino acid sequences, cDNA, and genomic DNA associated with each putative ABF or ABF were obtained from the Phytozome database, while we used the Simple Modular Architecture Research Tool (SMART; http://smart.emblheidelberg. de/smart/set_mode.cgi?NORMAL = 1) to identify ABFs with protein structures containing bZIP and other common domains. The Compute pI/Mw tool, from ExPASy (http://web.expasy.org/compute_pi/), was used to generate the theoretical molecular mass and Pi (isoelectric point) values.

### Phylogenetic tree construction

The TranslatorX server (http://translatorx.co.uk/) [46] was used to align the coding sequences (CDS), while we conducted a jModelTest analysis [47] to identify the model with the best fit. We created a ML (maximum likelihood) tree using the online program RAxML (http://www.trex.uqam.ca/index.php?action=raxml&project=trex) [48], via the best-fit model with 100 bootstrap samples. FigTree (http://tree.bio.ed.ac.uk/software/ figtree/) was used to visualize the phylogenetic tree.

### Analysis of gene structure analysis and conserved motif detection

The online Gene Structure Display Server (GSDS; http://gsds.cbi.pku.edu.cn) was used to assess the distribution of introns and exons and intron phase patterns. The Multiple Expectation Maximization for Motif Elicitation program (MEME; http://meme.nbcr.net/meme/cgi-bin/meme.cgi) was used to obtain the functional motifs of ABF proteins, using these parameters: maximum number of motifs = 20, optimum motif width = 6 to 100 residues, distribution of motifs = any number of repetitions.

### *ABF* gene expression profile

qRT-PCR was used to assess ABF expression patterns under different stress conditions, and TRIZOL reagent (Biotopped) was used to isolate total RNA using at least five seedlings from the three separate experiments. Total RNA was treated with Rnase-free DNAse (Takara) to remove genetic contamination. A Nanodrop1000 (Thermo Scientific product, USA) was used to measure the total RNA levels, while 5 μg of total RNA was run on 0.8% agarose gel from each sample to validate the number and integrity of RNA. The cDNAs were synthesized using recombinant M-MLV reverse transcriptase and total RNA (1 μg) mixed with 1 μg Oligo (dT)23 (Promega). The PCR conditions involved a preliminary denaturation for 10 min at 95 °C, 40 cycles of 15 s at 95 °C, and 40 cycles of 30 s at 60 °C. The internal control was *TaActin* (*TraesCS1A01G274400*). We applied the quantification method (2^-ΔCt^) and approximated the expression variation using three biological replicates [49]. Additional file 9: Table S2 outlines the primers used in this study.

### Subcellular localization of TaABF3-GFP fusion proteins

Prof. Zhensheng Kang’s Lab (Northwest A&F University, China) provided the *Triticum aestivum* cv. Chinese spring, which was used in the functional analysis of *TaABF3*. The full-length CDS sequence of *TaABF3* was amplified using PCR from the wheat *cv*. Chinese Spring with specific primers for the subcellular localization assay of TaABF3. This was then placed into the binary vector pCAMV35S::GFP, in between *BamH* I and *Xba* I, to find the subcellular localization of TaABF3. Sequencing was used to obtain positive clones, and the wheat mesophyll protoplasts were obtained from the constructs using the methods previously described [50]. A confocal microscope (Olympus, FluoViewTM FV300, Japan) was used to assess GFP fluorescence.

### Transformation of *Arabidopsis* and isolation of *TaABF3*

Prof. Zhensheng Kang’s Lab (Northwest A&F University, China) provided the *Arabidopsis* ecotype Columbia, which was used to transform *TaABF3*. We amplified the full-length opening reading frame of *TaABF3* from the wheat cv. Chinese Spring, using gene-specific primers which were subsequently cloned with the cauliflower mosaic virus (CaMV) 35S promoter into the pGreen0029-GFP vector. We then introduced the recombinant vector (*35S::TaABF3-GFP*) into *Agrobacterium tumefaciens*, and used the floral dip method [51] to transform it into *Arabidopsis* (*Arabidopsis thaliana*; ecotype Columbia). T_1_ seeds were placed on a MS medium containing 2% sucrose and 50 mg/mL kanamycin to identify the transformants. Phenotypic analyses were performed using homozygous T_3_ plants.

### Drought tolerance assay

We placed germinated, seven-day-old transgenic *Arabidopsis* plants on an MS medium into pots with a 130 g mix of 2:1 mixture of Jiffy mix and vermiculite to perform the drought tolerance assays. The 32-day-old plants grown under optimal conditions (22 °C, relative humidity of 60%, 16/8 h light/dark photoperiod) were subjected to drought stress conditions by withholding water from the plant for 14 days, after which they were watered and allowed to recover. We then counted how many plants survived after 6 days. A minimum of 48 plants from each line were analyzed against wild-type (WT) plants for each test. Statistical data shown is based on data obtained from the three independent experiments. We used a student’s *t*-test to analyze the differences between transgenic and WT plants.

### Measuring proline, MDA, and soluble sugar levels

We measured the proline contents, MDA levels, and soluble sugar levels of the transgenic and WT plants subjected to 10 days of drought stress, at which most leaves began to wilt, using detection kits (Solarbio) according to the manufacturer’s instructions.

### Statistical analyses

Each experiment was conducted a minimum of three times. Data shown are the mean ± standard deviation (SD) of the three independent replicates. A Student’s *t*-test was used to perform the statistical analysis, while *P* < 0.05 was considered statistically significant and *P* < 0.01 was considered extremely significant.

## Supplementary Information

**Additional file 1: Figure S1.** Phylogenetic relationship of group-A bZIP TFs from 29 plant species. The land plant group-A bZIP TFs are grouped into two major clades, designated as AREB/ABF and ABI5/AtDPBF subfamilies.

**Additional file 2: Table S1.** ABF protein properties and their clade-distributions in land plants. Gene identifiers are obtained from Phytozome database.

**Additional file 3: Figure S2.** Schematic diagram of gene structures of 95 plant *ABFs*. The thin lines represent introns and thick bars represent exons. The numbers above the gene structure indicate intron phases. A scale bar with a unit of base pair (bp) is graphed on the bottom.

**Additional file 4: Figure S3.** Alignment of 95 plant ABF protein sequences. The alignment was generated using ClustalW implemented in Geneious software and represented as thick lines (aligned characters) and thin lines (gaps). Overall alignment identity and a scale bar indicating the numbers of amino acid residues are graphed on the top.

**Additional file 5: Figure S4.** Combined motif diagram of 95 ABF proteins. Thick lines represent the ABF proteins. Different colored boxes represent separate and distinct motifs identified using MEME program. A scale bar indicating the numbers of amino acid residues is shown on the top. Motifs are drawn approximately to scale as boxes.

**Additional file 6: Figure S5.** Gene expression profile of *ABF* paralogs in plants. Gene expression data was extracted from *Arabidopsis thaliana (**http://jsp.weigelworld**. org/expviz/expviz.jsp)*, soybean (*Glycine max*, http://soybase.org/soyseq/), common bean (*Phaseolus vulgaris*, http://plantgrn.noble.org/PvGEA/SearchVisual.jsp), maize (*Zea mays*, http://www.plexdb.org/index.php), and rice (*Oryza sativa*, http://www.plexdb.org/index.php).

**Additional file 7: Figure S6.** Gene expression profile of *ABF* paralogs in *Arabidopsis thaliana* under different abiotic stresses. The mean-normalized expression values were obtained from the AtGenExpress microarray database via the web http://jsp.weigelworld.org/ expviz/expviz.jsp.

**Additional file 8: Figure S7.** RT-PCR analysis of *TaABF3* transcription levels in the transgenic *Arabidopsis* lines.

**Additional file 9: Table S2.** Primers used in this research.

## Data Availability

The datasets used and/or analysed during the current study are available from the corresponding author on reasonable request.

## Abbreviations

ABA: Abscisic acid
ABFs: Abscisic acid-responsive transcription factors
ABRE: ABA-responsive element
BLAST: Basic local alignment search tool
CDS: Coding sequences
DRE: dehydration-responsive element
GEO: Gene Expression Omnibus
GO: Gene Ontology
GSDS: Gene Structure Display Server
LCR: low complexity region
LTR: low temperature responsive element
MBS: MYB binding site involved in drought-inducibility
MEME: Multiple Expectation Maximization for Motif Elicitation
ML: Maximum likelihood
MYB: MYB recognition site
MYC: MYC recognition site
Pi: isoelectric point
SMART: Simple Modular Architecture Research Tool
TFs: Transcription factors
