# Supplementary figures and images for: Genome-wide analysis of the AREB/ABF gene lineage in land plants and functional analysis of TaABF3 in Arabidopsis
Source: BMC Plant Biol. 2020 Dec 10;20:558. doi: 10.1186/s12870-020-02783-9 (PMC7731569; doi:10.1186/s12870-020-02783-9)

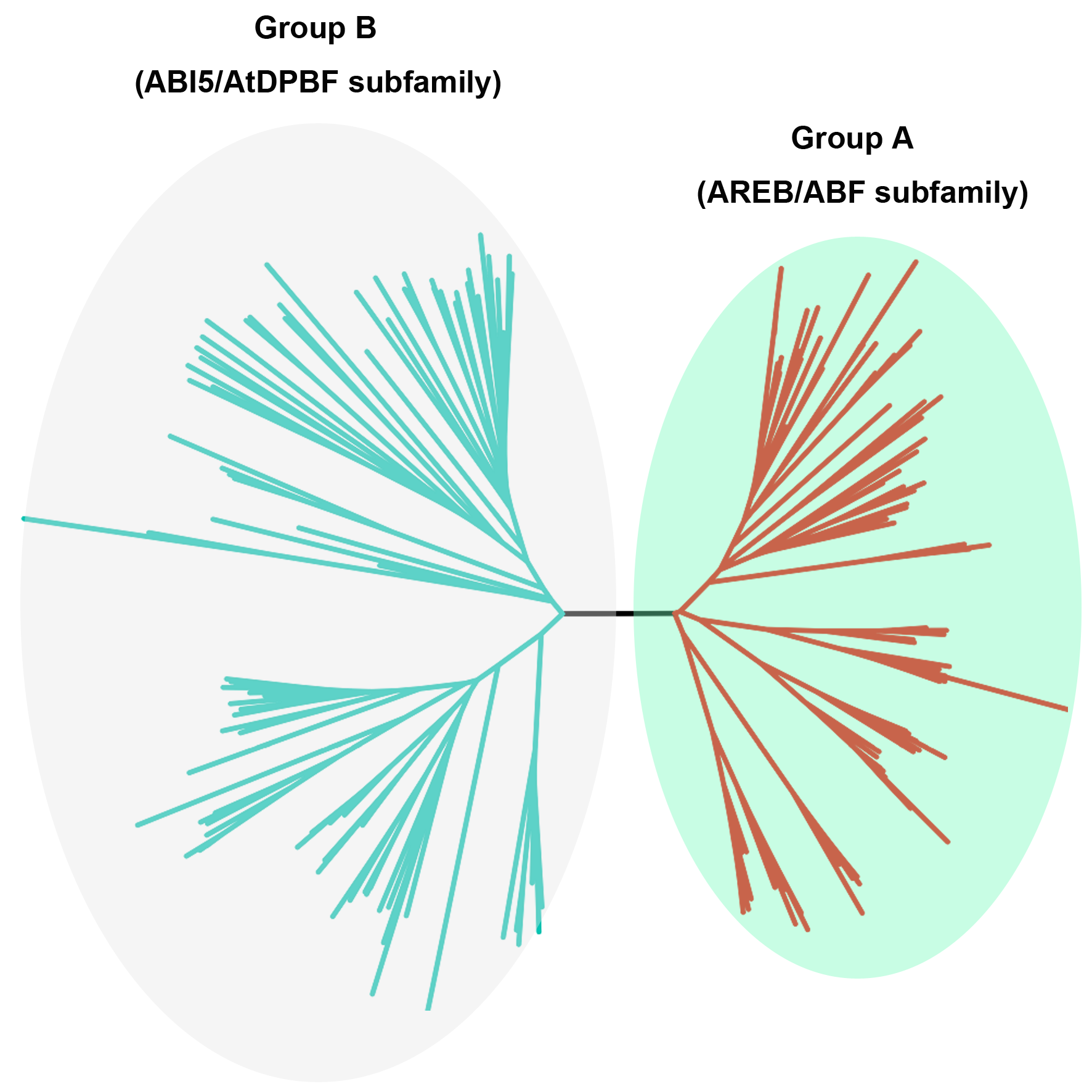

Supplement: Supplementary file 1 — Additional file 1: Figure S1. Phylogenetic relationship of group-A bZIP TFs from 29 plant species. The land plant group-A bZIP TFs are grouped into two major clades, designated as AREB/ABF and ABI5/AtDPBF subfamilies. [file 12870_2020_2783_MOESM1_ESM.tif]

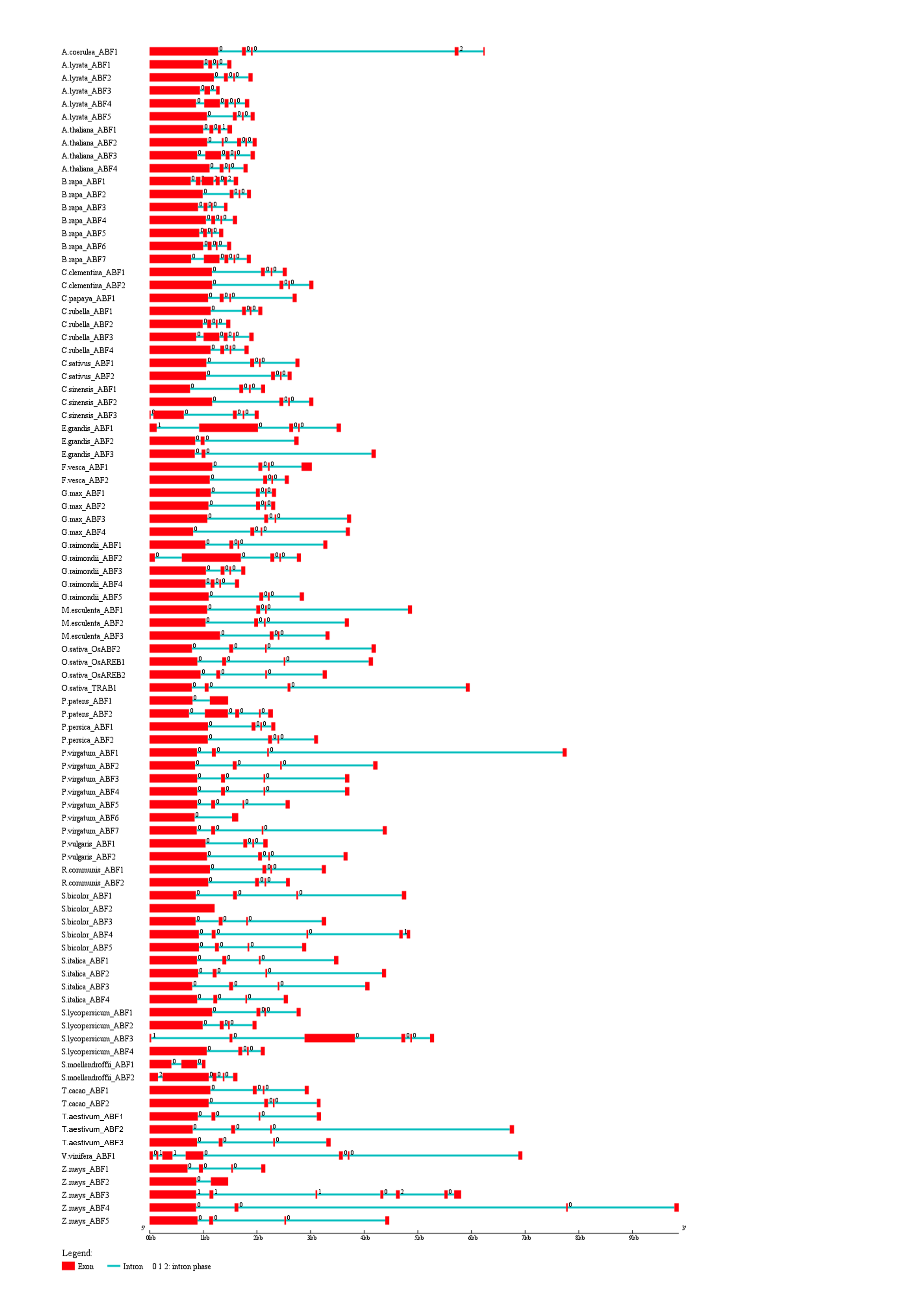

Supplement: Supplementary file 3 — Additional file 3: Figure S2. Schematic diagram of gene structures of 95 plant ABFs. The thin lines represent introns and thick bars represent exons. The numbers above the gene structure indicate intron phases. A scale bar with a unit of base pair (bp) is graphed on the bottom. [file 12870_2020_2783_MOESM3_ESM.tif]

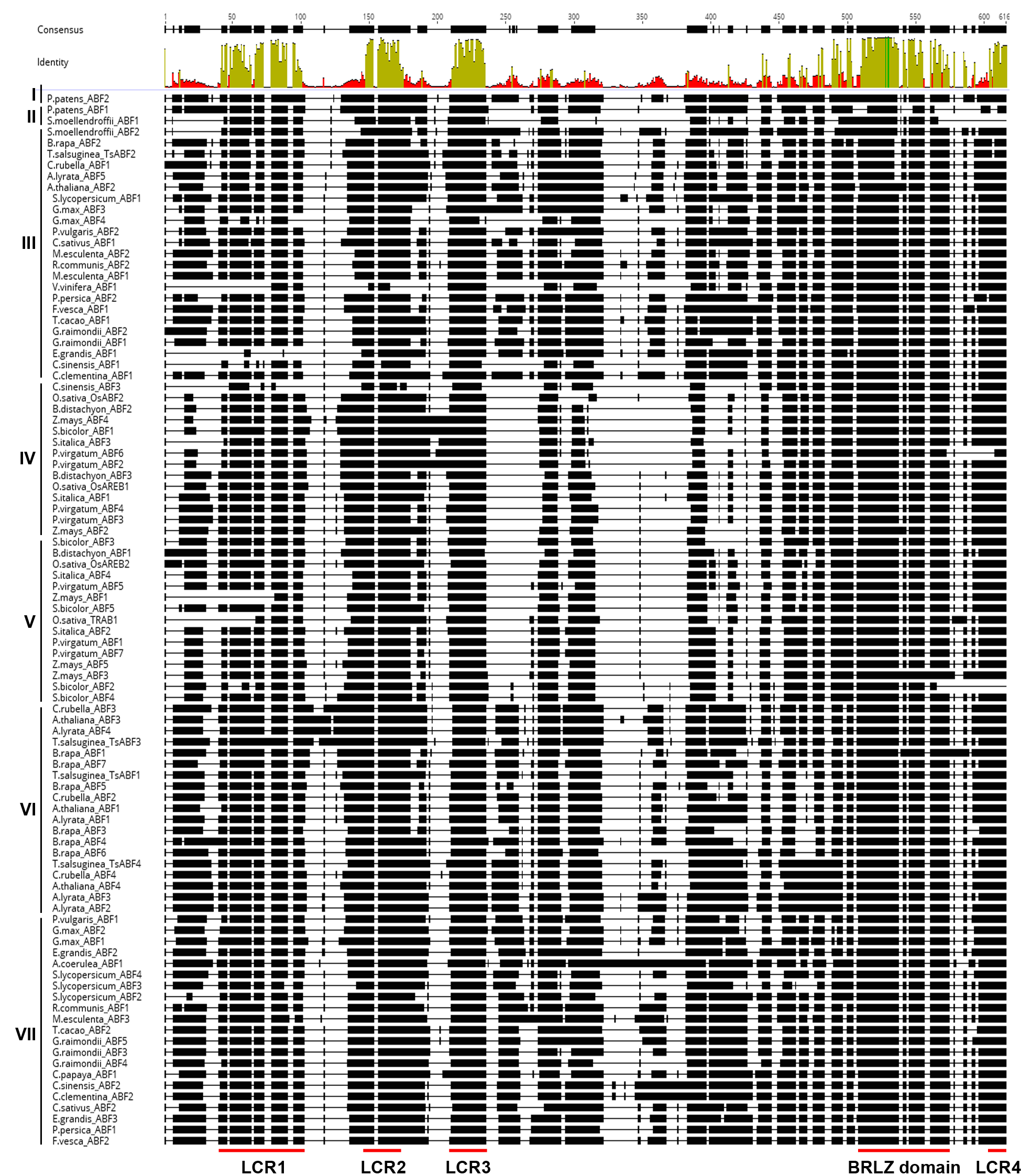

Supplement: Supplementary file 4 — Additional file 4: Figure S3. Alignment of 95 plant ABF protein sequences. The alignment was generated using ClustalW implemented in Geneious software and represented as thick lines (aligned characters) and thin lines (gaps). Overall alignment identity and a scale bar indicating the numbers of amino acid residues are graphed on the top. [file 12870_2020_2783_MOESM4_ESM.tif]

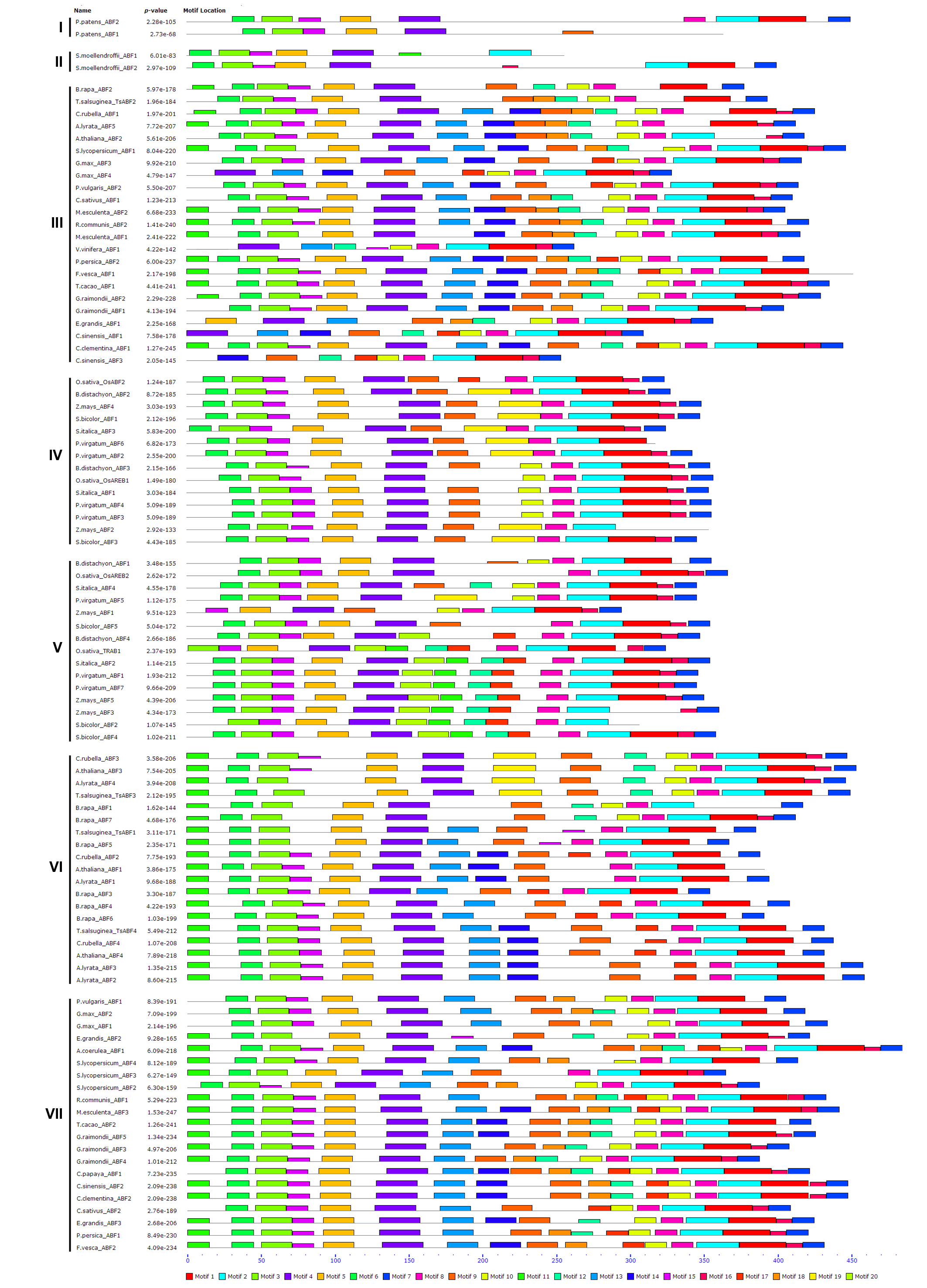

Supplement: Supplementary file 5 — Additional file 5: Figure S4. Combined motif diagram of 95 ABF proteins. Thick lines represent the ABF proteins. Different colored boxes represent separate and distinct motifs identified using MEME program. A scale bar indicating the numbers of amino acid residues is shown on the top. Motifs are drawn approximately to scale as boxes. [file 12870_2020_2783_MOESM5_ESM.tif]

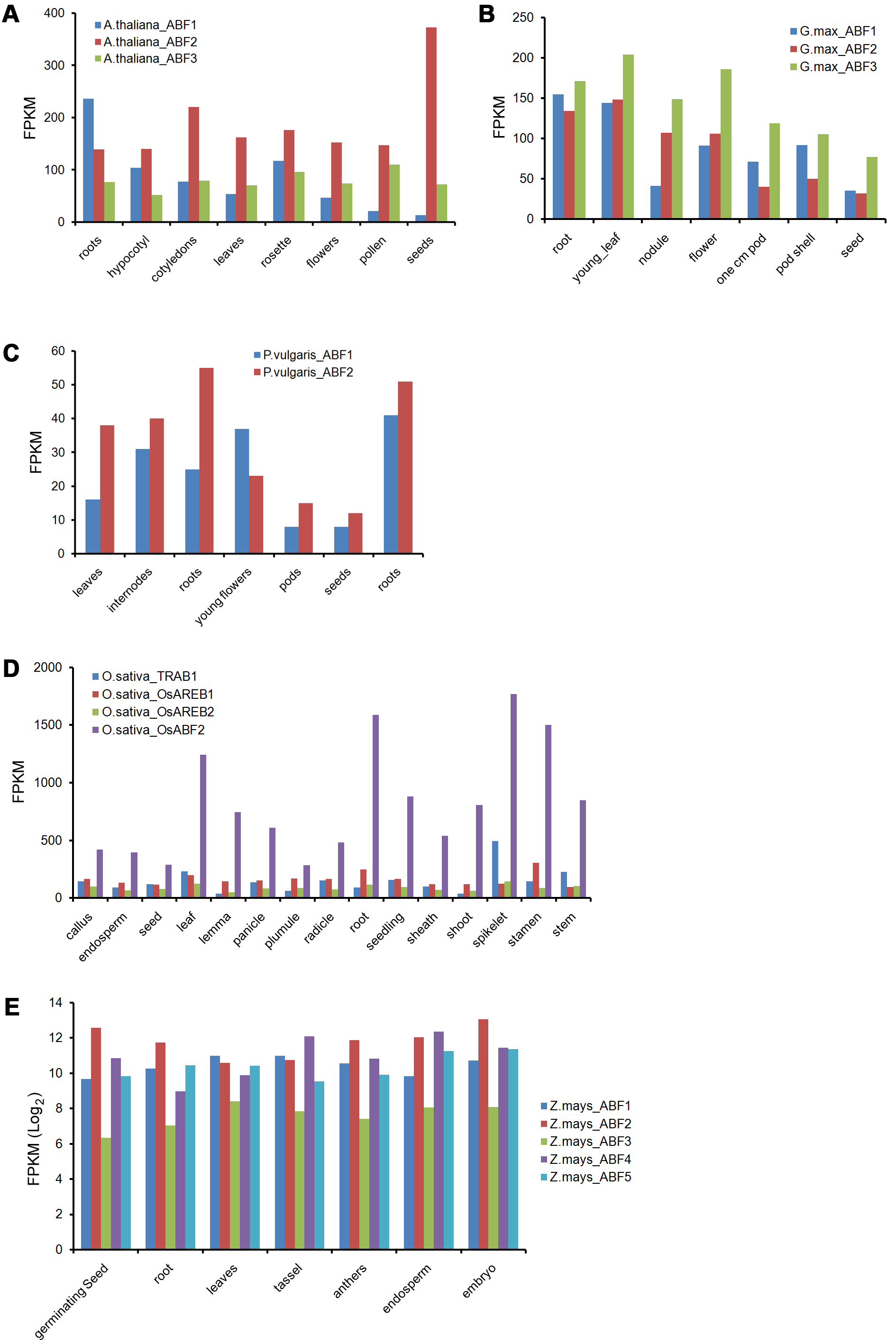

Supplement: Supplementary file 6 — Additional file 6: Figure S5. Gene expression profile of ABF paralogs in plants. Gene expression data was extracted from Arabidopsis thaliana (http://jsp.weigelworld. org/expviz/expviz.jsp), soybean (Glycine max, http://soybase.org/soyseq/), common bean (Phaseolus vulgaris, http://plantgrn.noble.org/PvGEA/SearchVisual.jsp), maize (Zea mays, http://www.plexdb.org/index.php), and rice (Oryza sativa, http://www.plexdb.org/index.php). [file 12870_2020_2783_MOESM6_ESM.tif]

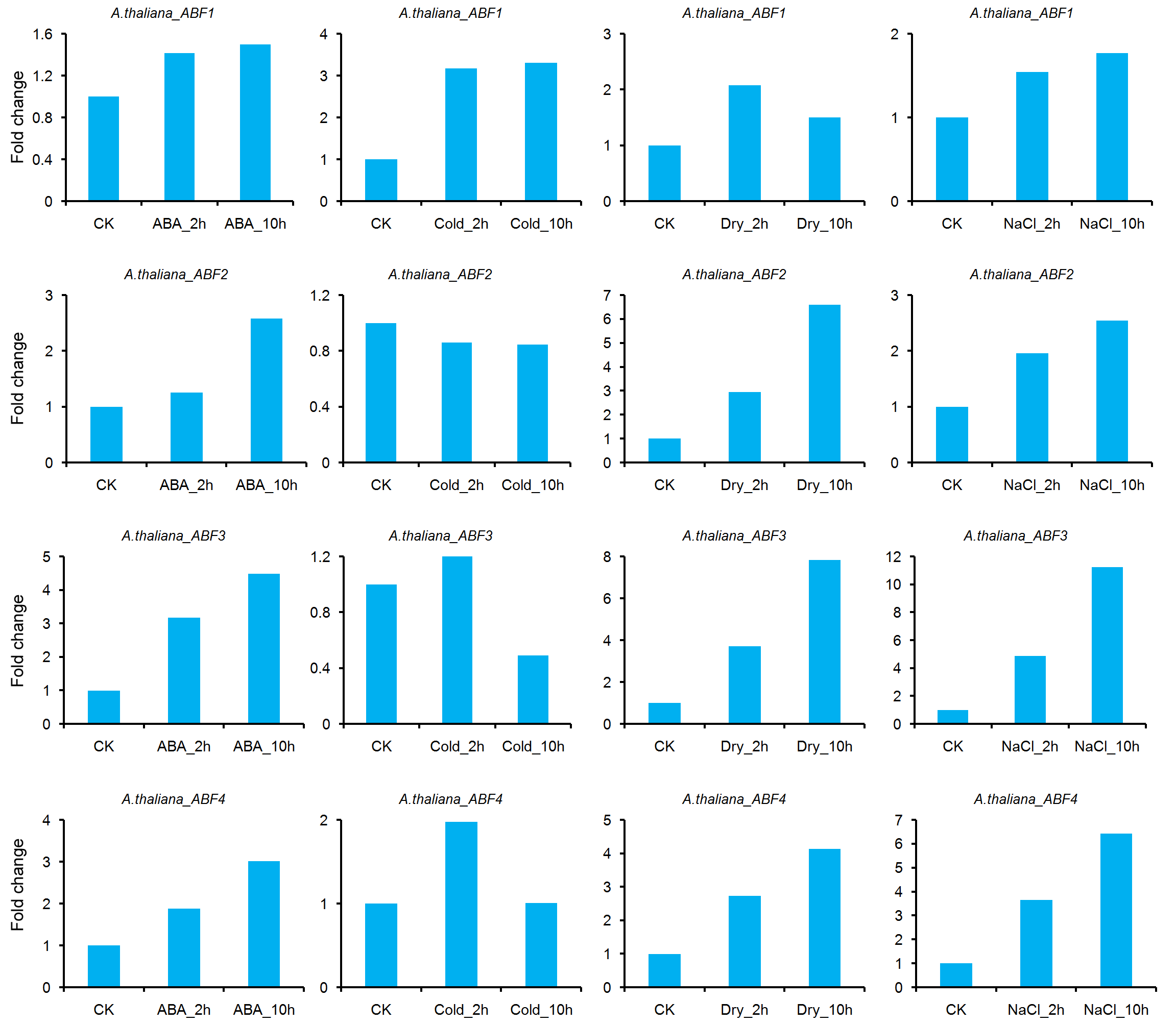

Supplement: Supplementary file 7 — Additional file 7: Figure S6. Gene expression profile of ABF paralogs in Arabidopsis thaliana under different abiotic stresses. The mean-normalized expression values were obtained from the AtGenExpress microarray database via the web http://jsp.weigelworld.org/ expviz/expviz.jsp. [file 12870_2020_2783_MOESM7_ESM.tif]

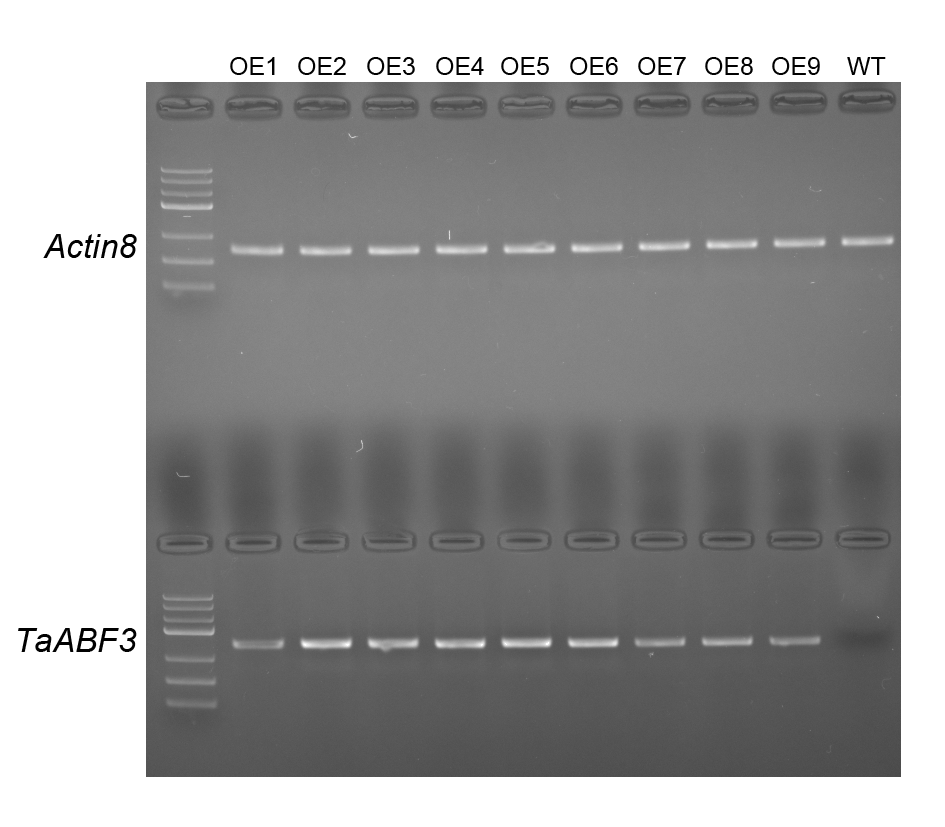

Supplement: Supplementary file 8 — Additional file 8: Figure S7. RT-PCR analysis of TaABF3 transcription levels in the transgenic Arabidopsis lines. [file 12870_2020_2783_MOESM8_ESM.tif]
